# Regulating response and leukocyte adhesion of human endothelial cell by gradient nanohole substrate

**DOI:** 10.1038/s41598-019-43573-0

**Published:** 2019-05-13

**Authors:** Li-Hua Huang, Long-Hui Cui, Dae Hwan Kim, Hyung Joon Joo, Ha-Rim Seo, Seung-Cheol Choi, Ji-Min Noh, Kyu Back Lee, Soon Jun Hong

**Affiliations:** 1 Department of Cardiology, Cardiovascular Center, Korea University Anam Hospital, 145, Anam-ro, Seongbuk-gu, Seoul 02841 Republic of Korea; 2 School of Biomedical Engineering, College of Health Science, Korea University, 145, Anam-ro, Seongbuk-gu, Seoul 02841 Republic of Korea

**Keywords:** Nanopores, Mechanotransduction

## Abstract

Understanding signals in the microenvironment that regulate endothelial cell behavior are important in tissue engineering. Although many studies have examined the cellular effects of nanotopography, no study has investigated the functional regulation of human endothelial cells grown on nano-sized gradient hole substrate. We examined the cellular response of human umbilical vein endothelial cells (HUVECs) by using a gradient nanohole substrate (GHS) with three different types of nanohole patterns (HP): which diameters were described in HP1, 120–200 nm; HP2, 200–280 nm; HP3, 280–360 nm. In results, HP2 GHS increased the attachment and proliferation of HUVECs. Also, gene expression of focal adhesion markers in HUVECs was significantly increased on HP2 GHS. *In vitro* tube formation assay showed the enhancement of tubular network formation of HUVECs after priming on GHS compared to Flat. Furthermore, leukocyte adhesion was also reduced in the HUVECs in a hole-diameter dependent manner. To summarize, optimal proliferations with reduced leukocyte adhesion of HUVECs were achieved by gradient nanohole substrate with 200–280 nm-sized holes.

## Introduction

Endothelial cells are the essential component of blood vessels which are in direct contact with vascular smooth muscles and connective tissues^1^. As endothelial cells located in the basement membrane, the endothelium acts as maintaining vessel homeostasis^2^. For example, during *in vitro* vessel remodeling, endothelial cells regulate smooth muscle cell stability through promoting the cell adhesion, attachment, and spreading during coculture of endothelial cells and smooth muscle cells^3^. Also, interactions of the endothelial cell to another cell or extracellular matrix (ECM) regulate intercellular signaling pathway in tight junction via adhesion molecules^4,5^. Besides, the intact structure and function of endothelium is vital in controlling the leukocyte extraction through blood vessels and platelet adhesion^6,7^. Damage to the endothelium could provoke various pathological changes in blood vessels such as endothelial-mediated inflammation, thrombosis, and vascular stenosis^8^. However, the biomimetic system for a functional understanding of endothelial cells was not well known yet.

Topographies of surrounding cells also play a crucial role in controlling cell functions. Especially, endothelial cells interact with ECM fibers via only one surface, and the fibrous scaffolds compose various sized nanohole structures^9^. The technology of nanopattern fabrication makes it possible to analyze and modulate cell behavior within 2-dimensional nano-scale topography. More importantly, recent reports have shown that the nano-scale surface topography plays a critical role in changing responses of various types of stem cells and adult cells, and for example, the mesenchymal stem cells (MSC) differentiation into osteoblasts or adipocytes was influenced by the substrate stiffness and nano-sized pattern^10^. Another study reported that nanopattern substrate could provide a regulatory signal to influence the human embryonic stem cell responses including cell morphology, adhesion, proliferation, and self-renewal potential^11^. Regarding endothelial cells, previous studies demonstrated that nanopattern substrate affected cell-to-matrix adhesion and cellular morphology^12^. Also, nanotopographies could increase cell-matrix adhesion, efficiently modulating endothelial cell functions by mechanotransduction^13,14^. However, no study has focused on response and leukocyte adhesion of endothelial cell within nanotopographical surfaces. Despite ongoing researches to elucidate the relationship between nanopattern substrates and cells^15^, there are still various limitations including difficulty in fabricating nanohole pattern dishes with various diameters. Also, it is the first time to adopt the gradient nanohole substrate (GHS) to screen the optimal growth condition for human umbilical vein endothelial cells (HUVECs), and GHS provides different conditions of nano-scale environment for evaluating the endothelial cell response.

In the present study, we used three different GHS with three type nanohole patterns (HP): HP1 with diameter 120–200 nm; HP2 with diameter 200–280 nm; HP3 with diameter 280–360 nm to evaluate cellular effects of HUVECs representing human endothelial cells. This study hypothesized that GHS would play a pivotal role in the HUVECs culture system and mimic endothelium microenvironment *in vitro*.

## Materials and Methods

### Fabrication of GHS

**Figure 1:**
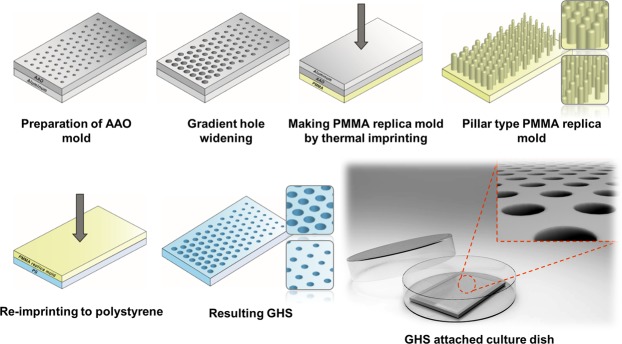
Schematic depiction of GHS fabrication.

Ultra-pure aluminum plate (99.999%), polymethylmethacrylate (PMMA) and polystyrene sheet were purchased from Goodfellow (UK). Chemicals used for the anodizing and hole-widening processes were provided by Samchun Chemical (South Korea). The anodic aluminum oxide (AAO) was fabricated as previously reported^16^. Illuminated by the multiple ultrastructure of *in vitro* ECMs, GHS was manufactured by using thermal nano-imprinting process (Fig. 1). The AAOs were gradually immersed in the etching solution (5.76 g phosphoric acid in 500 ml de-ionized water, 30 °C) using linear stage (Zaber, Canada) at a speed of 4.86 μm/sec for 2 hours. From this condition, the AAO mold for HP1 mold (120–200 nm) was fabricated. The AAO mold for HP2 (200–280 nm) and HP3 (280–360 nm) was fabricated by soaking the HP1 mold in the etching solution for another 2 or 4 hours. After hydroxylation by treating piranha solution (H_2_SO_4_:H_2_O_2_, 7:3 v/v), heptadecafluoro-1,1,2,2-tetrahydrodecyltrichlorosilane (HDFS, Gelest) self-assembled monolayers (SAMs) were formed on the surfaces of AAO molds by immersing in dehydrated n-hexane (Samchun Chemical) containing HDFS under a pure nitrogen atmosphere. After 10 minutes, HDFS SAMs covered AAO molds were sonicated in HFE-7100 (3 M) cleaning solution and completely dried in a vacuum desiccator for the following imprinting processes. AAO molds containing gradient nanometer-scale holes were thermally imprinted using a nano-imprinting device, NX-2000 (Nanonex Co.) on PMMA sheets. The PMMA sheets on which the AAO molds were mounted and transferred to a vacuum chamber of the device and thermally imprinted (190 °C, 300 psi) to form PMMA replica molds containing gradient nano-pillar arrays. When the samples were cooled to room temperature (RT), AAO mold, and PMMA replica mold were separated manually. Using the PMMA replica molds, polystyrene culture substrates containing gradient nanohole arrays were fabricated by thermal imprinting process (125 °C, 75 psi).

### *In vitro* culture of HUVECs

HUVECs (BioBud Inc.) were seeded on the Flat and GHS at a density of approximately 3 × 10^5^ of viable cells/substrate and maintained in EGM-2 MV (Lonza) medium supplemented with 5% fetal bovine serum, 100 U/ml of penicillin, and 50 U/ml of streptomycin in a 37 °C humidified incubator with 5% CO_2_. Culture medium was replaced every 2 days.

### Immunofluorescence staining

For immunofluorescence staining, HUVECs (5 × 10^3^ cells/cm^2^ or 1.5 × 10^4^ cells/cm^2^) were cultured for 48 hours on Flat and GHS. Samples were fixed with 2% paraformaldehyde (PFA) and were blocked with 5% normal goat serum in PBST (0.1% TritonX-100 in PBS) for 30 minutes at RT. Next, HUVECs were incubated for 2 hours at RT with primary antibodies: anti-human Vinculin antibody (Sigma), anti-human phosphohistone H3 (PHH3) antibody (Millipore). After washing with PBST for 3 times, samples were incubated for 1 hour with secondary antibodies at RT in the dark: Alexa flour-594-conjugated anti-mouse IgG (Invitrogen) and Alexa Fluor-488-conjugated Phalloidin (Invitrogen). The nuclei were stained with 4′ 6-diamidino-2-phenylindole (DAPI, Invitrogen) at RT in the dark, followed by mounting samples in fluorescent mounting medium (DAKO). Fluorescence images were acquired using confocal fluorescence microscope (Zeiss LSM 700, Carl Zeiss) and TE-FM Epifluorescence System attached to an Olympus BX61 inverted microscope. The number of PHH3 positive cells were quantified with Image-Pro plus 7.0 software. For calculation of PHH3 positive HUVECs, random sections of fluorescence images of PHH3-stained HUVECs grown on Flat, HP1, HP2 and HP3 GHS were analyzed. For visualization of focal adhesion (FA), Vinculin accumulated plaque was quantified. The “analyze particles” tool in Image J (NIH) was used to quantify the number of FA per cell and the averaging area of FA. At least 50 cells were analyzed for FA size and density quantification.

### Nucblue live cell staining

HUVEC (1.5 × 10^4^ cells/cm^2^) were seeded in each group. Visualization of attached HUVECs on Flat and GHS was achieved by fluorescence staining with loading solution containing EGM-2 MV medium and Nucblue live ready probes live reagent (Life technologies). After samples incubation for 20 minutes at 37 °C, loading solution was replaced by fresh EGM-2 MV. Attachment capacity of HUVECs was assessed at 6 hours based on fluorescence images that were acquired using the Leica optical microscope (Leica DMI3000B). Fluorescence and phase-contrast images were taken using a Canon EOS-600 digital camera. Quantification was performed using Image-Pro plus 7.0 software. Five randomized sections of images were quantified.

### Terminal deoxynucleotidyl transferase-mediated deoxyuridine triphosphate nick-end labeling (TUNEL) assay

For the assessment of apoptosis between HUVECs (1.5 × 10^4^ cells/cm^2^) were cultured on the Flat and GHS, the experiment was performed using the APO-BrDU TUNEL assay kit (Invitrogen), according to the manufacturer’s instructions. TUNEL activity in cells was determined by immunofluorescence staining. Each sample was fixed with 2% PFA and incubated with the DNA-labeling solution for 1 hour at 37 °C. Alexa Fluor 488-dye-labeled anti-BrdU antibody was used to stain the nuclei, and then propidium iodide/RNase A staining buffer was added to each sample. Following incubation, samples were mounted in the fluorescent mounting medium. Fluorescence images were acquired using TE-FM Epifluorescence System attached to an Olympus BX61 inverted microscope. Quantification of TUNEL positive cells was performed with Image-Pro plus 7.0 software.

### *In vitro* tube formation assay

*In vitro* tube formation assay was performed as described previously^17^. Briefly, 300 µl of growth factor reduced Matrigel matrix (BD Biosciences) was applied to each well of a 24-well plate at 37 °C for 1 hour for gelling. HUVECs were trypsinized and 3 × 10^4^ cells/well were reseeded onto Matrigel-coated wells at 37 °C after culturing on the Flat and GHS for 48 hours. The forming tube structure of spread cells was observed in employing a Leica microscope and acquired Canon EOS-600D digital camera at 1, 3 and 24 hours. Branching point and tube forming area of HUVECs were quantified with Image-Pro plus 7.0 software. Branching point was defined as three overhanging branches of intersection point on Flat and GHS.

### Peripheral blood mononuclear cells (PBMNCs) isolation and spreading in HUVECs

PBMNCs were freshly isolated from adult whole peripheral blood samples of healthy donors, applying the Ficoll-isolation (GE-Healthcare) procedure^18,19^. Following separation of PBMNCs, cells were stained with Nucblue live ready probes live reagent. Then 3 × 10^5^ of cells/substrate of PBMNCs were plated on HUVECs cultured for 48 hours on Flat and GHS. EGM-2 MV medium was changed once after 3 hours. Fluorescence images were acquired employing Leica optical microscope at 3, 6 and 24 hours. The number of adherent PBMNCs was assessed by Image-Pro plus 7.0 software. For quantification of adherent PBMNCs, five randomized fluorescence images of spreading PBMNCs were analyzed.

### Cellular RNA preparation and quantitative polymerase chain reaction (PCR)

Total RNAs was extracted from HUVECs with Trizol (MRC) according to the manufacturer’s instructions. 500 ng of RNA was reverse-transcribed into complementary DNA using M-MLV reverse transcriptase (Invitrogen). Quantitative PCR used iQ SYBR Green Supermix (Bio-Rad), and indicated primers were performed using MyiQ2 detection system (Bio-Rad). Relative gene expression levels were quantified based on the delta Ct and normalized to the reference gene GAPDH. Table 1 shows primers used for quantitative PCR. Measurement of gene expression was assayed in triplicate.

**Table 1:** Summary of quantitative PCR primers.

Gene name	Forward sequence (5′-3′)	Reverse sequence (5′-3′)	Product (bp)
GAPDH	GAGTCCACTGGCGTCTTCAC	TTCACACCCATGACGAACAT	119
VCL	GATGAAGCTCGCAAATGGTC	TCTGCCTCAGCTACAACACCT	77
TLN1	ACCAGTGACTATGGCCGTCT	CGGTGTTTGATATGGGAACC	89
PXN	CAGCAGACACGCATCTCG	GAGCTGCTCCCTGTCTTCC	107
ITGA2	GCTGATAATTTGGTCAACCTCA	GAACATTCCCATCCGAAGAG	109
ITGA6	TTTGAAGATGGGCCTTATGAA	CCCTGAGTCCAAAGAAAAACC	102
ITGAV	GCACCCTCCTTCTGATCCT	GAGGACCTGCCCTCCTTC	113
ITGB1	CGATGCCATCATGCAAGT	AGTGAAACCCGGCATCTG	95
ITGB3	GCCCTGCTCATCTGGAAAC	TACAGTGGGTTGTTGGCTGT	110
ANGPT1	GGGGGAGGTTGGACTGTAAT	AGGGCACATTTGCACATACA	362
TIE2	GGACCTGAATGCAACCATCT	TTCACAAGCCTTCTCACACG	121
CXCR4	CCTGCCTGGTATTGTCATCC	AGGATGACTGTGGTCTTGAGG	105
vWF	TAAGTCTGAAGTAGAGGTGG	AGAGCAGCAGGAGCACTGGT	109
MCP-1	AGTCTCTGCCGCCCTTCT	GTGACTGGGGCATTGATTG	93
VCAM-1	TGCACAGTGACTTGTGGACAT	CCACTCATCTCGATTTCTGGA	92
ROCK1	CAGAAACTAGAACATTTGACTGGAAA	GCTCCAGTTGCAGGGTTAGA	76
ROCK2	ATAGCCCCTGGGTGGTTC	CCATTTTTCAGGCACATCATAA	128

### Western blotting

The HUVECs on Flat, HP1, HP2, and HP3 GHS for 2 days were washed twice with PBS and disrupted with 1 × cell lysis buffer (9803, Cell Signaling Technology) containing 1 mM phenylmethylsulfonyl fluoride (P7626, Sigma). A quantitative analysis of the samples was performed using the Bradford assay dye reagent (500–0006, Bio-Rad). The sample protein (10 μg) was boiled in 1 × loading dye for 5 min and subjected to electrophoresis in a 10% polyacrylamide gel with sodium dodecyl sulfate. After transfer to a polyvinylidene fluoride membrane (ISEQ. 00010, Millipore), the membranes were blocked with 5% bovine serum albumin containing 1 × TBST (a mixture of Tris-buffered saline and Tween 20; WH400028806, 3 M) at RT for 1 h. The membranes were incubated with anti-ROCK1 (1:1000; ab45171, Abcam), anti ROCK2 (1:1000; ab71598, Abcam), and anti-GAPDH (1:2000; G8795, Sigma) antibodies at RT for 2 h. The membranes were then washed three times with TBST and incubated with a horseradish peroxidase-conjugated secondary antibody (1:3000; Santa Cruz) in TBST at RT for 1 h. Chemiluminescence was visualized with the ECL Plus reagent (32132, Thermo Fisher Scientific) and recorded on X-ray film.

### Measurement of inflammatory cytokine levels

To measure the Interleukin-6 (IL-6; D6050) and interleukin-8 (IL-8; D8000C), HUVECs (4.3 × 10^4^ cells/cm^2^) were seeded in Flat, HP1, HP2, and HP3 GHS and cultured in EGM-2 medium for 24 hours. Then the level of IL-6 and IL-8 in the culture medium was measured by ELISA (R&D systems, Minneapolis, MN).

### Statistics

All statistical values presented were expressed as mean ± standard (SD) deviation of the mean. Significant differences between means were determined by ANOVA Student–Newman–Keuls test. ^*^p < 0.05 was statistically significant. Statistical analysis was performed using Sigma Stat 3.5 software (SPSS, IL, USA).

## Results

### Basement membrane of endothelial cells and characterization of GHS

**Figure 2:**
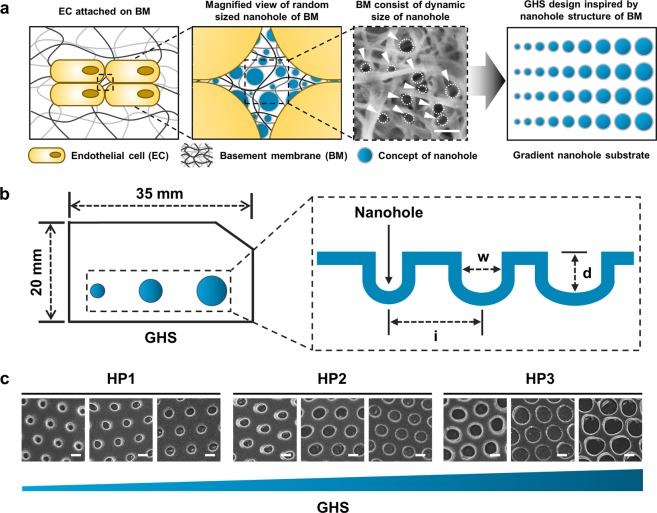
Characteristics of GHS. (**a**) Platform diagram of endothelial cell attached in BM and GHS design inspired by nanohole structure of BM. Dashed white circles indicate the nanohole structure of BM. (**b**) Illustration of GHS. Diameter range (w) of GHS are 120–200 nm, 200–280 nm, and 280–360 nm, respectively. The depths (**d**) and intervals (i) of all GHS are consistent of 440 nm. (**c**) Representative SEM images of HP1, HP2, and HP3 GHS. Scale bars are 200 nm.

Endothelial cells adhered to the basement membrane via only one surface (Fig. 2a). As can be seen from the illustration of GHS, the structures were considerably replicated with integrity and consistency over an area of 35 × 20 mm^2^ (Fig. 2b). Representative SEM images confirmed that substrates consisted of intended size-gradient nanoholes (Fig. 2c).

### HP2 GHS significantly increase the focal adhesion response of HUVECs

**Figure 3:**
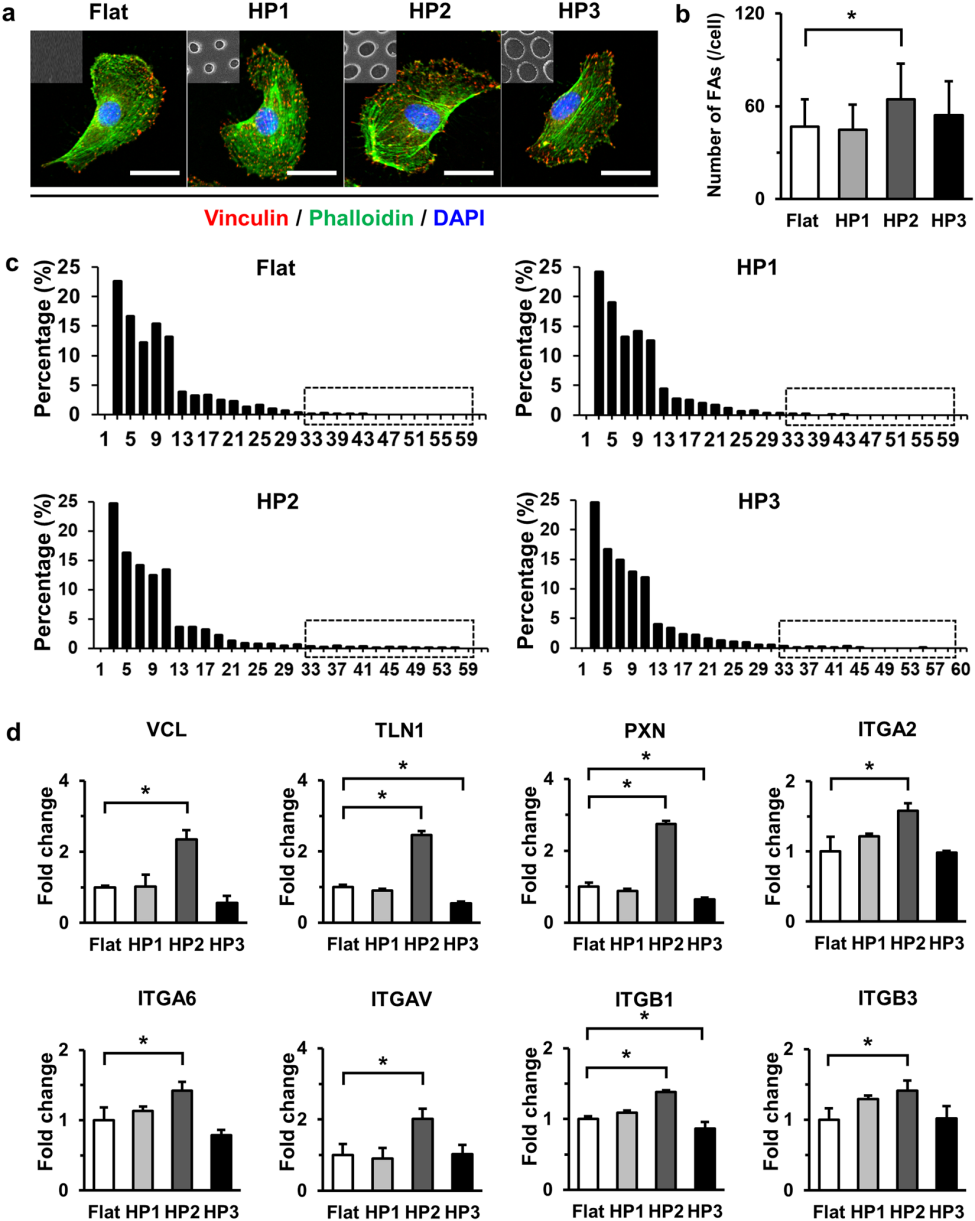
Comparison of focal adhesion response between Flat and GHS. (**a**) Representative immunofluorescence images of vinculin (red), phalloidin (green) and DAPI (blue) of HUVECs on Flat and GHS. Scale bars are 10 μm. (**b**) Quantification of the number of vinculin accumulated plaque per cell on Flat and GHS (n = 55). ^*^p < 0.05. (**c**) Histogram showed the frequency distribution of FA. Percentages of FA areas formed in HUVECs cultured on Flat and GHS were quantified. Dashed rectangle indicates that FA area range between 30 and 60 μm^2^. (**d**) Quantitative PCR analysis of the relative expression of focal adhesion markers. ^*^p < 0.05.

We studied the effect of nanohole topography on FA number and area using quantification of vinculin expression in HUVECs on Flat and GHS. FA formed on GHS were distributed throughout the cytoplasm and cell periphery while FAs generated on Flat were mostly located on the cell periphery (Fig. 3a). The number of FA in HUVECs grown on HP2 GHS was significantly higher compared to that of Flat and HP2 GHS has sustaining increased trend of vinculin intensity, indicating that the FA number was affected by the nanohole diameters (Fig. 3b). The area of FA distribution was observed to extend up to 59 μm^2^, and the tightest distribution was found on HP2 GHS. However, FAs were rarely observed on Flat and HP1 GHS beyond an area of 45 μm^2^ (Fig. 3c). Gene expression levels of focal adhesion markers in HUVECs were assessed on Flat and GHS. Expressions of VCL, TLN1, PXN, ITGA2, ITGA6, ITGAV, ITGB1 and ITGB3 in HUVECs cultured on HP2 GHS were significantly increased than that of Flat. (Fig. 3d).

### HP2 GHS significantly increase the attachment and proliferation potency of HUVECs

**Figure 4:**
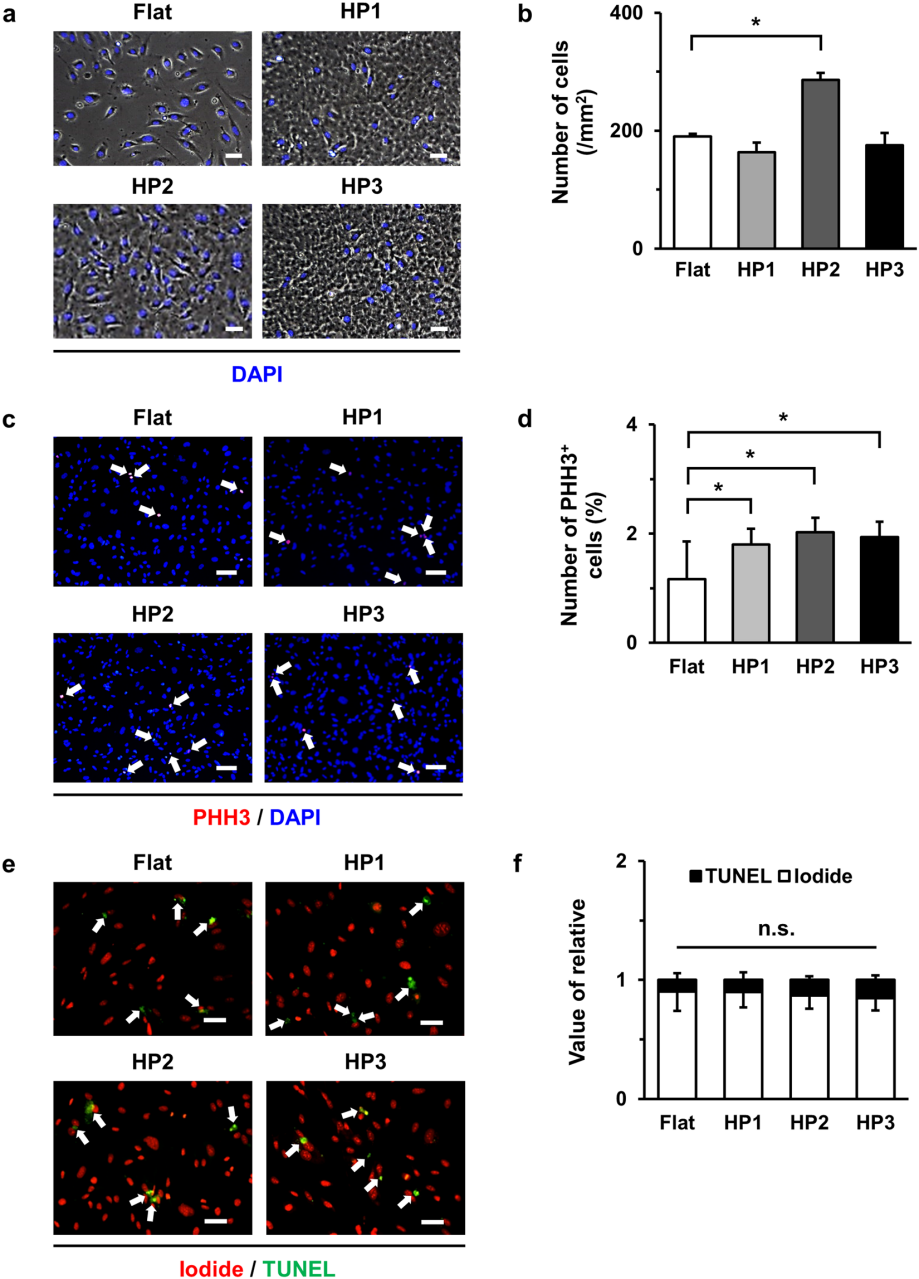
Viability of HUVECs on Flat and GHS. (**a**) Representative images showed that attachment of HUVECs on Flat and GHS after culturing for 6 hours. Scale bars are 50 μm. (**b**) Quantification of the number of attached HUVECs. ^*^p < 0.05. (**c**) Proliferation images of HUVECs on Flat and GHS were shown by immunofluorescence staining with PHH3 (red) and DAPI (blue). White arrows indicated PHH3 positive HUVECs. Scale bars are 100 μm. (**d**) Quantitative analysis of proliferation rate of HUVECs. ^*^p < 0.05. (**e**) Representative immunofluorescence images of HUVECs cultured on Flat, and GHS were shown. Live cells were marked with iodide (red), and dead cells were labeled as TUNEL (green; see white arrows). White arrows indicated TUNEL positive HUVECs. Scale bars are 50 μm. (**f**) Quantitative analysis of apoptosis rate of HUVECs. *n*.*s*. = non-significant variables.

The attachment rate was observed at 6 hours after seeding. The results revealed that the number of attached HUVECs on HP2 GHS was significantly higher than that of Flat (Fig. 4a,b). Proliferation in HUVECs on GHS was also significantly greater compared to that of Flat after seeding at 48 hours (Fig. 4c,d). However, differences in apoptosis rates of HUVECs on GHS and Flat were not observed (Fig. 4e,f). Therefore, the result showed that HP2 GHS increased attachment and proliferation of HUVECs compared to Flat, but apoptosis rate of HUVECs was not affected by GHS.

### Branching point and tube area significantly increasing in HUVECs cultured on HP2

**Figure 5:**
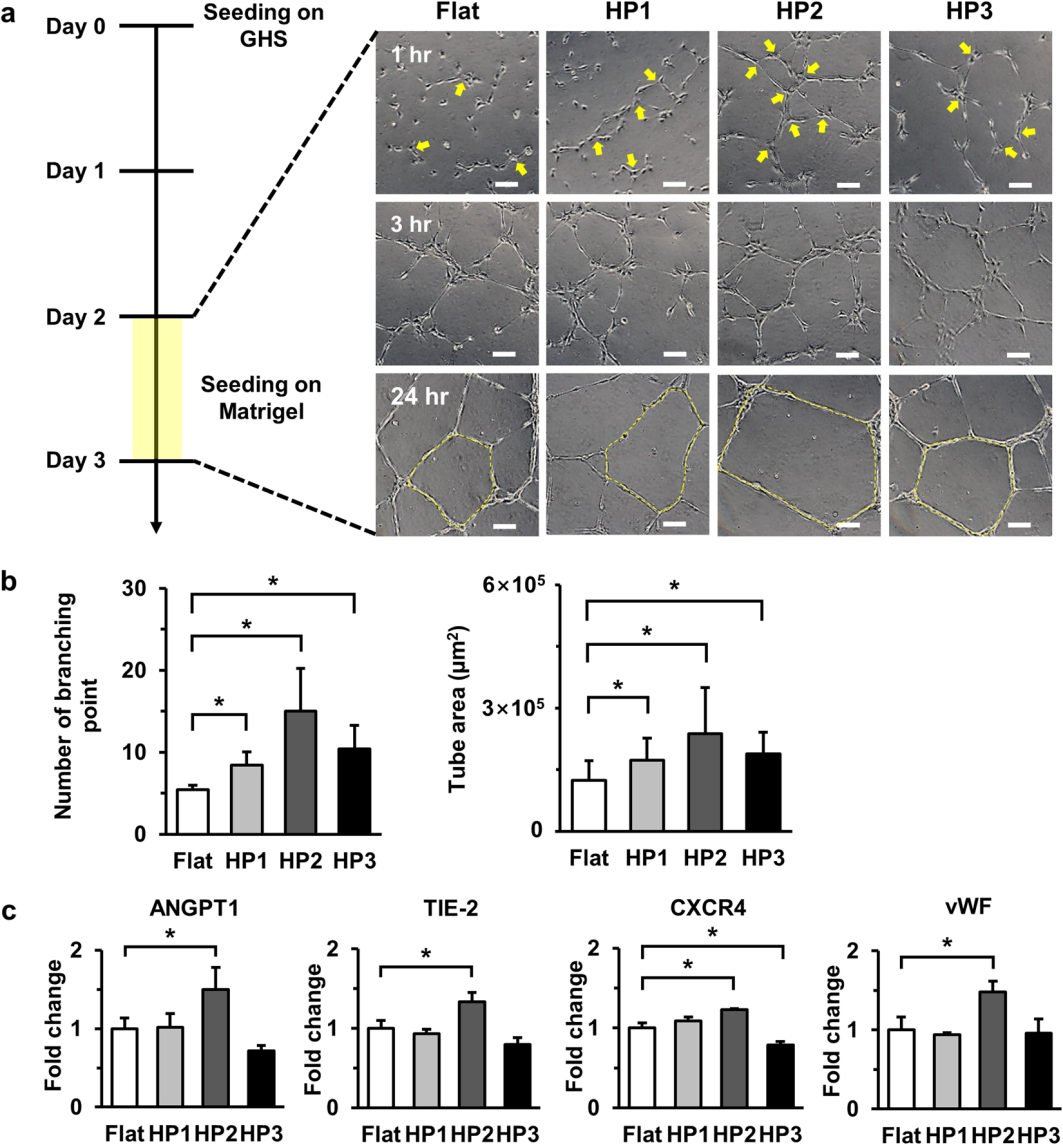
HUVECs stimulated by HP2 GHS rapidly induces tube formation. (**a**) *In vitro* tube forming images of HUVECs cultured on Matrigel for 1, 3 and 24 hours were shown. Yellow arrows indicated branching points of HUVECs at 1 hour. Yellow dotted lines were tubular borders at 24 hours. Scale bars are 200 μm. (**b**) Quantitative analysis of the number of branching points and tube forming areas formed at 1 hour and 24 hours after seeding respectively. ^*^p < 0.05. (**c**) Quantitative PCR data showed expressions of ANGPT1, TIE-2, CXCR4 and vWF for vascular endothelial cells. ^*^p < 0.05.

Functional ability of HUVECs on the Flat and GHS was evaluated by *in vitro* capillary tube formation on Matrigel. HUVECs after 48 hours of cultivation on Flat and GHS were detached and reseeded on Matrigel. After seeding on Matrigel, images at 1, 3, and 24 hours were obtained (Fig. 5a). *In vitro* tube formation assay revealed that HUVECs stimulated by GHS revealed a significantly greater number of branching points at 1 hour than Flat. However, mature tube structures at 24 hours were found in HUVECs stimulated by HP2 GHS (Fig. 5b). Expressions of ANGPT1, TIE-2, CXCR4, and vWF were significantly increased in HUVECs grown on HP2 GHS (Fig. 5c). Also, the ROCK gene and protein expression showed an increased trend in HUVECs cultured on HP2 GHS (Supplementary Figs 1, 2).

### HP2 significantly decrease the leukocyte adhesion and MCP-1, VCAM-1 gene expression

**Figure 6:**
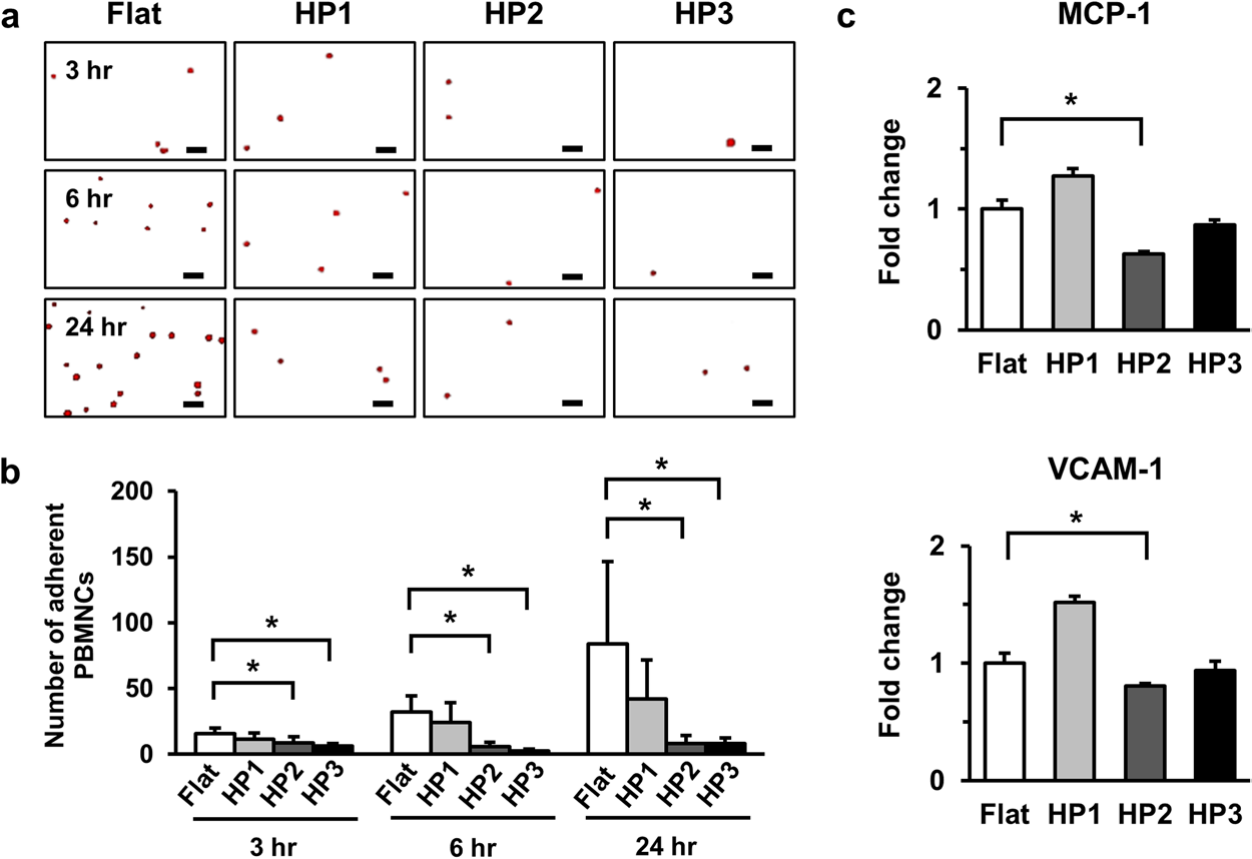
Synergistic effects of HUVECs and GHS interaction with leukocyte adhesion. (**a**) Representative images of adherent PBMNCs spread on HUVECs after 3, 6 and 24 hours were shown. PBMNCs were stained with Nucblue (red). Scale bars are 50 μm. (**b**) Quantification of PBMNCs adhesion rates at 3, 6 and 24 hours on Flat and GHS. ^*^p < 0.05. (**c**) qPCR data showed expressions of MCP-1 and VCAM-1. ^*^p < 0.05.

To assess responses of HUVECs grown on Flat and GHS, PBMNCs were isolated and spread on HUVECs (Fig. 6a and Supplementary Fig. 3). The adhesion of PBMNCs to HUVECs stimulated by HP2 and HP3 GHS was significantly reduced compared to those of Flat at 3, 6 and 24 hours after PBMNC seeding (Fig. 6b). Also, gene expression of MCP-1 and VCAM-1 was significantly lower on HP2 GHS compared to that of Flat (Fig. 6c). Proinflammatory cytokine IL-8 of HUVECs was significantly decreased on HP2 GHS (Supplementary Fig. 4).

## Discussion

This study revealed that; (i) HUVECs showed the highest attachment and proliferation capacity on HP2 (200–280 nm) GHS, (ii) HP2 GHS was determined to be the optimal diameter for extending greater tube forming potential to HUVECs and (iii) HP2 GHS reduce the leukocyte adhesion compared to Flat. Designing tools from the concept of “cell niche” have been emerging as a novel strategy for observation of cell viability as well as exchanging cell character and function^20^. Also, a previous study showed that nanogrooves with 1:1, 1:3, and 1:5 spacing ratio (width:spacing, width = 550 nm) could control the adhesion, migration, and differentiation of human MSCs^21^. In the present study, we for the first time attempted the fabrication of gradient-sized nanohole substrate (hole diameter was 120 nm to 360 nm) to explore the optimal nanohole diameter for cellular response and cell function in HUVECs.

In our previous study, we also demonstrated that the response and focal adhesion distribution of endothelial colony-forming cells changed by the specific sized nanopillar surface^22,23^. In the present study, the HP2 GHS showed the significant increase in the number of FA (Fig. 3b) and the integrin-related gene expressions (Fig. 3d). While, the attachment and proliferation of HUVECs on HP2 GHS were significantly higher than Flat, and HUVECs on GHS showed a similar apoptosis rate compared to that of Flat (Fig. 4a–d). The ROCK1 and ROCK2 gene expression significantly increased in HP2. Also, the ROCK protein expression revealed an increased trend in HP2 GHS. The ROCK inhibitor assay data shows nanohole stimuli mainly through the ROCK signaling, and it may influence the cytoskeleton reorganization of HUVECs (Supplementary Figs 1, 2). In other words, the specific interval size of nanohole could modulate the attachment potential of HUVECs, and these changes may affect the cell proliferation and *in vitro* tube formation. The improvement of cell viability seems to be strongly associated with cues of GHS.

FA, known as cell-matrix adhesions, are large, integrin-containing, multi-protein assemblies spanning the plasma membrane that link the cellular cytoskeleton to surrounding extracellular matrix^24^. Vinculin, a component of focal adhesion as well as Talin, Paxillin and Integrin family, plays a vital role in maintaining attachment to extracellular matrix^25^. A previous study reported that FA density and size were higher on a modified topography compared to that on unpatterned control^26^. Increase of FA number in HUVECs grown on HP2 GHS suggests that physical cues from GHS could lead to the changes of FA formation. However, FA area of HUVECs on GHS was similar to that of Flat. The current results showed that HP2 GHS provided strong physical stimulation to HUVECs, which influenced the vinculin plaque number. Generally, a lower number of FA was believed to promote cell migration rates^27,28^. Based on previous reports, a greater number of FA localization throughout cell cytoplasm on GHS could be regarded as upregulating the stability of cell adhesion. Although the relationship between FA number and cell stability in endothelial cells on GHS was not fully addressed, FA number might modulate cell stability through stimulation of GHS.

HUVECs induced network formation at 6 hours after seeding on Matrigel and maintained their network formation until 24 hours. In tube formation assay, the number of branching points in HUVECs at 1 hour was significantly higher on HP2 GHS, and tube formation was maintained for 24 hours. This result suggested that the increase of branching point in HUVECs could indicate rapid tube formation. Further studies to elucidate the association with stimulated HUVECs and GHS would be necessary although the formation of tube area was the largest on HP2 GHS. Importantly, we observed that ANGPT1, TIE-2, CXCR4 and vWF expressions gradually increased on HP2 GHS (Fig. 5c), suggesting that upregulation of angiogenesis related genes might lead to rapid tube formation. Previous reports suggested that ANGPT1/TIE2 pathway was crucial for maintaining the physical interaction with endothelial or smooth muscle cells and was important for prevention of cell death^29,30^. Therefore, an increase of TIE-2 expression in HUVECs cultured on HP2 GHS could be involved in physical interaction with nanotopography.

The adhesion of leukocytes to vascular endothelium was known to a hallmark of the inflammatory process^31,32^. Leukocyte adhesion was closely related to endothelial activation and infiltration to injured tissue^33,34^. Based on previous reports, PBMNCs were isolated and spread in HUVECs on Flat and GHS to identify the effect of GHS on leukocyte adhesion on HUVECs. PBMNCs on HUVECs, which were cultured on HP2 and HP3 GHS, showed the lower attachment rate compared to that of Flat. The MCP-1 and VCAM-1 expression in HUVECs cultured on HP2 was significantly decreased compared to that of Flat (Fig. 6c). The previous report demonstrated that MCP-1 triggered firm adhesion of monocytes to vascular endothelium^35^. Another study reported that increased binding of adhesion molecules such as VCAM-1 to its ligand on leukocytes was regulated by the number of molecules expressed on the endothelial cells and by conformational changes that occur with cellular activation^36^. Furthermore, the proinflammatory cytokine IL-8 of HUVECs on HP2 was significantly decreased than those of HUVECs on Flat (Supplementary Fig. 4). These results indicate that HUVECs on HP2 GHS could reduce leukocyte adhesion via stabilization of quiescent status. Therefore, GHS-coated stents could be a useful strategy in reducing the inflammatory process after the vascular intervention, and clinical trials with GHS-coated stents would be warranted.

**Figure 7:**
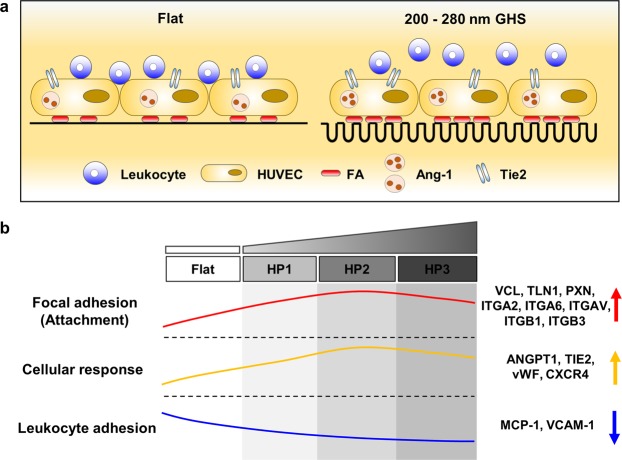
Diagram representing the responses of HUVECs to GHS. (**a**) An illustrative summary of cellular responses of HUVECs on GHS. (**b**) Relative trends of focal adhesion, cellular response, and leukocyte adhesion in HUVECs on Flat and GHS.

Based on experimental observations, our study showed that how GHS surface could control the regulation of HUVECs in each step: attachment, proliferation, and endothelial function according to respective substrates. Futhermore, HP2 GHS strongly influenced endothelial cell attachment, proliferation, *in vitro* tube formation, and leukocyte adhesion (Fig. 7). We expected that biophysical cues from the GHS would be key parameters for controlling the activation or quiescence of HUVECs. Our results clarified the mechanotransductive roles of GHS to regulate the cellular responses of HUVECs via FA formation, which led to endothelial cell activation or quiescence.

## Conclusion

In conclusion, GHS has ability to enhance the attachment, proliferation, tube formation of HUVECs. Especially, the attachment rate of PBMNCs on HUVECs layer grown on HP2 (200 nm–280 nm) GHS was significantly lower than that of Flat. Furthermore, nanohole stimuli could affect integrin related gene (VCL, TLN1, PXN, ITGA2, ITGA6, ITGAV, ITGB1, and ITGB3), focal adhesion protein (vinculin), angiogenesis related gene (ANGPT1, TIE-2, CXCR4, and vWF), inflammatory related gene (MCP-1 and VCAM-1), and proinflammatory cytokine (IL-8). In addition, nanohole stimuli may through the ROCK signaling to modulate the HUVECs behaviour. These results suggest that HUVECs sensitive to nanohole size of substrate and provide the possibility of applying nano interface-based implant devices in various diseases. Controlling the response of endothelial cells using GHS could be useful tools in the field of cardiovascular regenerative therapy.

## Supplementary information

Supplementary Information
