# Supplementary material for: Regulating response and leukocyte adhesion of human endothelial cell by gradient nanohole substrate
Source: Sci Rep. 2019 May 13;9:7272. doi: 10.1038/s41598-019-43573-0 (PMC6514209; doi:10.1038/s41598-019-43573-0)
Supplement: Supplementary file 1 — Supplementary Information [file 41598_2019_43573_MOESM1_ESM.docx]

**Regulating response and leukocyte adhesion of human endothelial cell by gradient nanohole substrate**

Li-Hua Huang^1, *^, Long-Hui Cui^1, *^, Dae Hwan Kim^2, *^, Hyung Joon Joo^1^, Ha-Rim Seo^1^, Seung-Cheol Choi^1^, Ji-Min Noh^1^, Kyu Back Lee^2, #^ and Soon Jun Hong^1, #^

^1^ Department of Cardiology, Cardiovascular Center, Korea University Anam Hospital, 145, Anam-ro, Seongbuk-gu, Seoul, 02841, Republic of Korea

^2^ School of Biomedical Engineering, College of Health Science, Korea University, 145, Anam-ro, Seongbuk-gu, Seoul, 02841, Republic of Korea

^*^ These authors equally contributed to this work

^#^ These authors equally supervised this work

**Running title**

HUVECs on gradient nanohole substrate

**Address correspondence to**

^#^ Soon Jun Hong, M.D., Ph.D., Professor

Department of Cardiology, Cardiovascular Center, Korea University Anam Hospital, 145, Anam-ro, Seongbuk-gu, Seoul, 02841, Republic of Korea

Phone: +82-2-920-5445; Fax: +82-2-927-1214; E-mail: psyche94@gmail.com

and

^#^ Kyu Back Lee, M.D., Ph.D., Professor

School of Biomedical Engineering, College of Health Science, Korea University Anam Hospital, 145, Anam-ro, Seongbuk-gu, Seoul, 02841, Republic of Korea

Phone: +82-2-3290-5655; Fax: +82-2-929-8044; E-mail: kblee@korea.ac.kr

**Supplementary Figure 1**


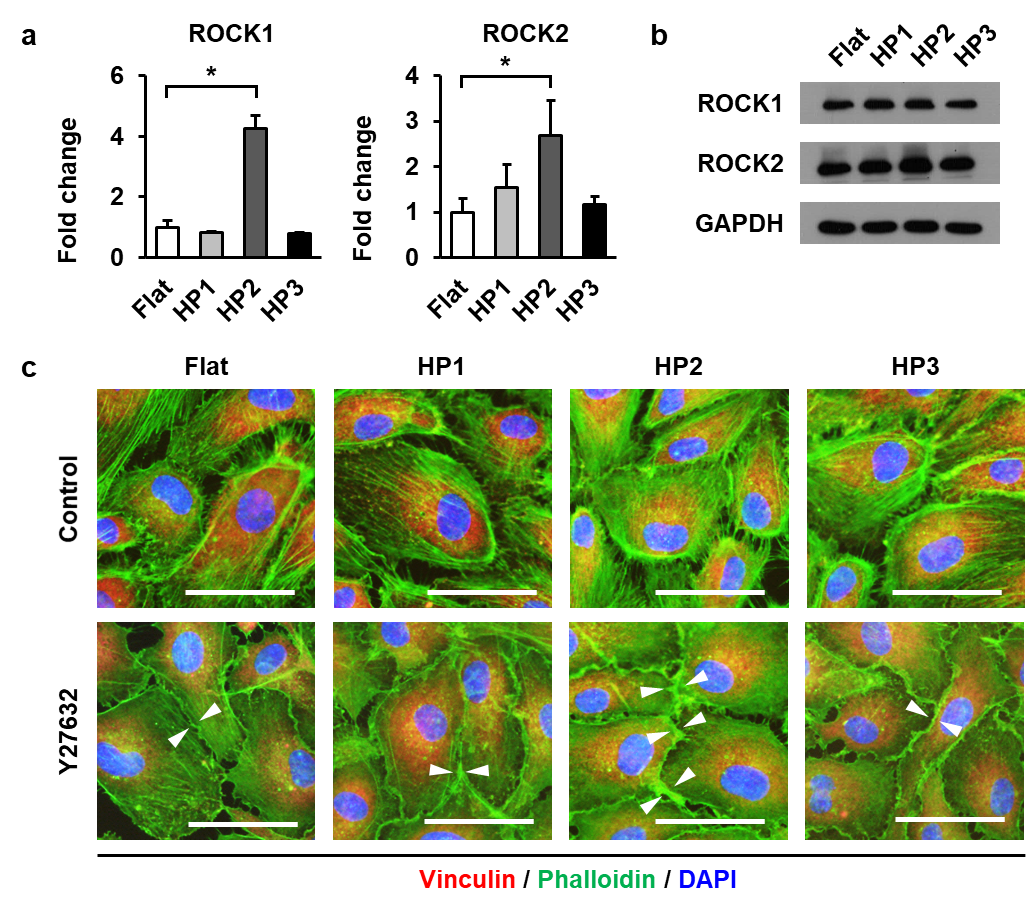


**Supplementary Figure 1.** Impairment of the nanohole stimuli from GHS by suppression of ROCK. (A) qPCR analysis of the expression of ROCK1 and ROCK2 in HUVECs cultured on Flat, HP1, HP2, HP3 for 2 days. Three independent experiments were conducted; **p* < 0.05 (B) Representative western blotting images of ROCK1 and ROCK2 expression in HUVECs cultured on Flat (lane 1) and on HP1 (lane 2), HP2 (lane 3), and HP3 (lane 4) GHS for 2 days. GAPDH served as an endogenous control. (C) Representative immunofluorescent images showing F-actin (green) and vinculin (red) and DAPI-stained cell nuclei (blue) in HUVECs cultured on Flat or GHS without (-Y27632) or with (+Y27632; 10 μmol) the ROCK inhibitor. White arrows indicate edge part of cytoskeleton in HUVECs. Scale bars are 50 μm.

**Supplementary Figure 2**


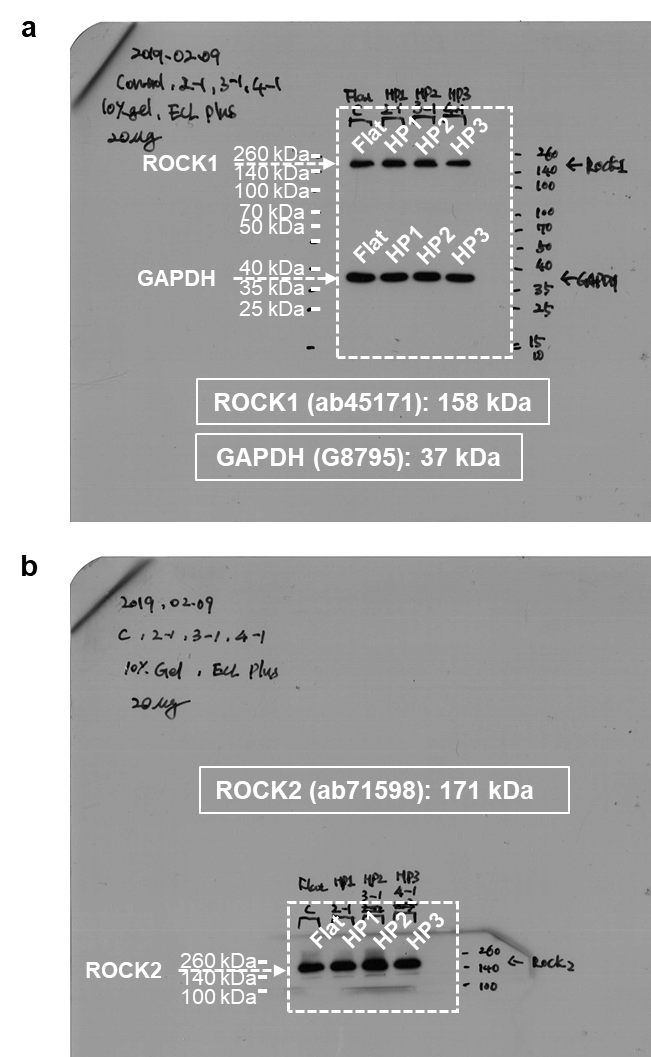


**Supplementary Figure 2.** Original western blotting image of (a) ROCK1, GAPDH and (b) ROCK2. Dashed rectangle indicates cropped area for supplementary Figure 1b.

**Supplementary Figure 3**


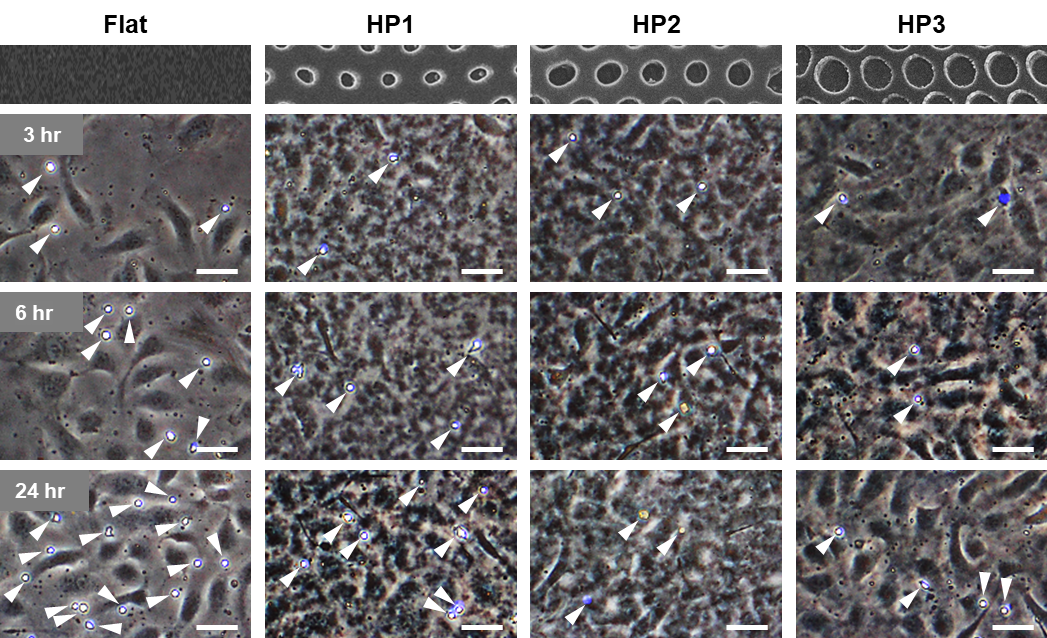


**Supplementary Figure 3.** Representative images of adherent PBMNCs spread on HUVECs after 3, 6 and 24 hours were shown. PBMNCs were stained with Nucblue (blue). White arrows indicate attached PBMNCs. Scale bars are 50 μm.

**Supplementary Figure 4**


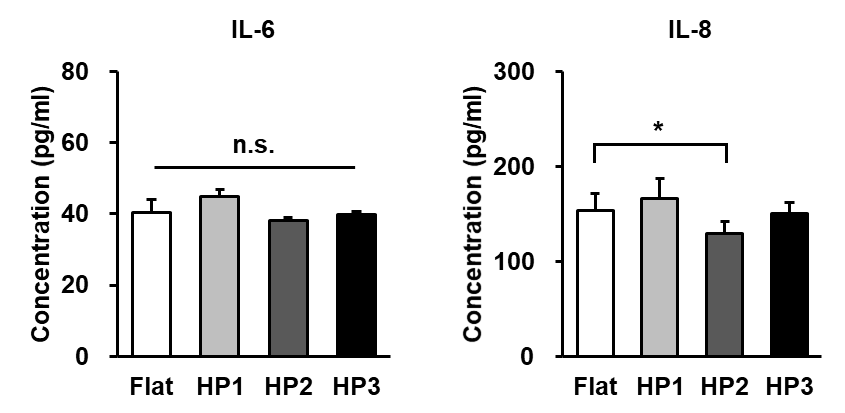


**Supplementary Figure 4.** ELISA analysis of HUVECs cultured on the Flat, HP1, HP2, and HP3 GHS. Quantification data of released IL-6 and IL-8 in HUVECs were cultured on Flat and GHS after 2 days. ^*^*p* < 0.05.
